# Analysis of Beef Peptonoids

**Published:** 1884-06

**Authors:** John Attfield

**Affiliations:** London


### Analysis of Beef Peptonoids.

Following is a report on Beef Peptonoids by Prof. John Attfield, F. R. S., F. I. C., etc., author of "A Manual on Chemistry, General, Medical and Pharmaceutical."

"The chemical examination to which I have submitted your Beef Peptonoids yields the following results in 100 parts—

| | |
|---|---|
| Albuminoids (containing nitrogen 10.94), - | 69.25 |
| Fat, - - - - - | 10.71 |
| Sugar, including a trace of starch, - - | 9.50 |
| Phosphates, equal to bone phosphate, - | 3.01 |
| Other mineral substances, - - - | 2.61 |
| Moisture, - - - - | 4.92 |
| | 100.00 |

"The manufacturers of Beef Peptonoids state that this food is composed of dry, lean beef, one-third; the solids of milk, minus most of the fat, one-third; the gluten of wheat, one-third; the beef being partially digested or "peptonized." My analysis fully supports this statement, for I find present between 69 and 70 per cent. of albuminoids, that is, flesh-forming material (nitrogen 10.94); more than 20 per cent. of warmth-producing substance, nearly half of which is milk sugar, and rather more than half fat; three per cent. of bone-forming phosphates; about two per cent. of other normal mineral matter, and about five per cent. of moisture. A sample of the constituent gluten submitted to me was practically pure, containing a mere trace of starch. Rather more than one-fourth of the albuminoids, probably the "peptonized" portion, was soluble; while practically the whole of the Beef Peptonoids was readily soluble in peptonizing fluids, showing that it is easily and wholly digested when taken into the stomach. The flavor and odor of the preparation are excellent; its thorough state of dryness fits it for keeping any length of time in any climate. It is by far the most nutritious and concentrated food I have ever met with. Indeed, a palatable and assimilable and in every way acceptable article of food, containing nearly 70 per cent. of truly nutritive nitrogenous material partially peptonized has never before, to my knowledge, been offered to the medical profession or to the public."

LONDON, Nov. 18, 1883. JOHN ATTFIELD.

